# Supplementary material for: A Nuclear Magnetic Resonance (NMR)- and Mass Spectrometry (MS)-Based Saturation Kinetics Model of a Bryophyllum pinnatum Decoction as a Treatment for Kidney Stones
Source: Int J Mol Sci. 2024 May 12;25(10):5280. doi: 10.3390/ijms25105280 (PMC11121557; doi:10.3390/ijms25105280)
Supplement: Supplementary file 1 [file ijms-25-05280-s001.zip › ijms-2962489-supplementary.pdf]

Supplementary Figures:

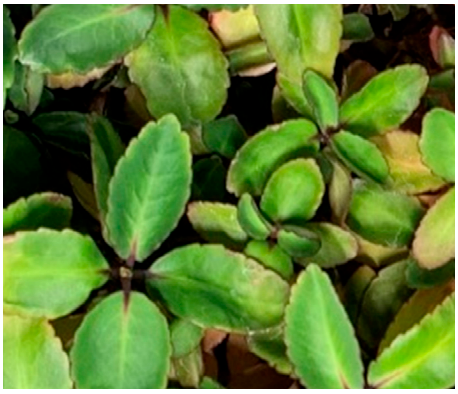

Figure S1. *Bryophyllum pinnatum* plant growing indoor at the Faculty of Agricultural and Environmental Sciences, McDonald Campus, McGill University.
